# Nasal Obstruction—I

**Published:** 1908-06-20

**Authors:** 


### Laryngology and Rhinology.

### NASAL OBSTRUCTION—I.

The troubles which are the result of nasal obstruction are of a most varied and far-reaching character. The effect of obstruction in early life in modifying the shape and development of the bones of the face and jaws was described in The Hospital of Dec. 21, 1907. In the present article other effects of nasal insufficiency will be considered.

The most obvious effect of nasal obstruction is mouth-breathing. The lining membrane of the nose is extremely well supplied with glands and blood-vessels, by which means it can not only saturate the inspired air with aqueous vapour, but is able to keep itself thoroughly moistened while performing this task. When, however, the air is inspired through the open mouth, the mucous membranes of the tongue, palate, and pharynx have to discharge this function as well as they can. Under ordinary circumstances they are able to saturate the air with moisture for a short time, but they sacrifice themselves in doing so and rapidly become dry; the air is no longer moistened when it reaches the larynx, and this organ has to take up the duty, and so the dryness spreads. In addition to moistening the air the nose also warms it, and, what is still more important, filters it from dust and micro-organisms. By the time it reaches the naso-pharynx air inspired through the healthy nose is almost free from germs. It is no wonder, then, that catarrh of the pharynx, larynx, and bronchi are frequent results of nasal obstruction. Dryness of the mouth and throat, especially on waking in the morning, is an almost constant symptom, but patients vary very much in the degree to which this affects them; in some the fact is only elicited by questioning, in others the discomfort is almost intolerable. It is well-recognised that laryngitis is very largely the result of nasal obstruction; in addition to the effects of the unfiltered air, the deficient nasal resonance throws an added strain on the larynx in producing the voice, and thus increases the tendency to inflammation. In cases of chronic bronchitis and winter cough, nasal obstruction is often the determining factor, but more important than all these is the question of the part played by nasal insufficiency in the ætiology of pulmonary phthisis. There is evidence to show that infection does not commonly occur by direct inhalation into the lungs, but rather by the entrance of bacilli through the faucial lymphatic ring, and thence by the cervical lymph channels to the apex of the lung. In either case it is clear that tubercle bacilli can hardly enter by inhalation through a healthy nose, but can readily gain admittance when an individual breathes through the open mouth. The view that mouth-breathing predisposes to phthisis, and that the normal nose is a valuable protection, is becoming widely accepted and is borne out by the large proportion of cases of nasal obstruction to be found among consumptive patients. Additional support is afforded by the fact that patients with atrophic rhinitis, whose noses have lost their filtering function, are decidedly liable to phthisis.

Another class of the ill-effects of nasal obstruction appears to be due to increase of the inspiratory negative pressure behind the obstruction. At first sight it appears a simple matter to assume that there is in such cases an increased negative pressure, or suction, during inspiration, and that the effects are due to this, but there are several objections to this assumption. The pigeon-breast and in-drawn ribs frequently seen in cases of adenoids may be explained in this way; but when the adenoid hypertrophy is so considerable as to produce constant mouth-breathing the suction effect might be expected to disappear, and yet the deformity of the chest occurs. Again, retraction of the membrana tympani is common in cases of nasal obstruction, and has been explained in the same way, but with the same objection. Here the result is probably due rather to catarrh spreading to the Eustachian tube from the seat of the obstruction. In cases of adenoids, there is rarely mechanical blocking of the tube by the growth, but frequently catarrhal inflammation of the tube and middle ear. When the septum is deflected, the ear on the narrow side is generally the worse and is often the only one affected, and this can hardly be explained by the theory of suction. The parts behind the obstruction are often in a state of swelling and catarrh, which is probably due rather to the absence of the normal current of air and to obstructed drainage of the mucous secretion, and this in turn increases the obstruction. After removal of the mechanical obstruction the soft parts tend to regain their normal condition.

A third group of symptoms seem to result from insufficient oxygenation of the blood. Especially during sleep, when the tongue drops back on to the pharynx, a very considerable degree of asphyxiation takes place, and the patient wakes unrefreshed, has a dull headache in the morning, and suffers from general lassitude and ill-health; children often show, in addition, marked failure of growth.

Finally, certain symptoms are due to local pressure and irritation within the nose. Although nasal obstruction is not necessarily present, they may be mentioned here for the sake of completeness, for they are caused by the nasal abnormalities which produce obstruction. In this connection one of the most important is enlargement of the middle turbinal body of one or both sides, which becomes nipped between the septum and outer wall. If the enlargement be unilateral it pushes the septum over, and so may compress the turbinal of the opposite side. This causes pain and a feeling of pressure over the bridge of the nose, often frontal headache and inability to concentrate the attention. Some of the severest cases of supraorbital neuralgia owe their origin to this cause, though removal of the enlarged turbinal will not always effect a permanent cure. A septal spur, which is so large as to impinge on the outer wall of the nose, may cause similar symptoms or may be the starting point of various reflex neuroses, such as paroxysmal sneezing and asthma.

